# Novel Approaches to Detect Serum Biomarkers for Clinical Response to Interferon-β Treatment in Multiple Sclerosis

**DOI:** 10.1371/journal.pone.0010484

**Published:** 2010-05-05

**Authors:** Kaushal S. Gandhi, Fiona C. McKay, Eve Diefenbach, Ben Crossett, Stephen D. Schibeci, Robert N. Heard, Graeme J. Stewart, David R. Booth, Jonathan W. Arthur

**Affiliations:** 1 Westmead Millennium Institute, University of Sydney, Sydney, Australia; 2 Discipline of Medicine, Sydney Medical School, University of Sydney, Sydney, Australia; 3 School of Molecular and Microbial Bioscience, University of Sydney, Sydney, Australia; 4 Sydney Bioinformatics, University of Sydney, Sydney, Australia; INMI, Italy

## Abstract

Interferon beta (IFNβ) is the most common immunomodulatory treatment for relapsing-remitting multiple sclerosis (RRMS). However, some patients fail to respond to treatment. In this study, we identified putative clinical response markers in the serum and plasma of people with multiple sclerosis (MS) treated with IFNβ. In a discovery-driven approach, we use 2D-difference gel electrophoresis (DIGE) to identify putative clinical response markers and apply power calculations to identify the sample size required to further validate those markers. In the process we have optimized a DIGE protocol for plasma to obtain cost effective and high resolution gels for effective spot comparison. APOA1, A2M, and FIBB were identified as putative clinical response markers. Power calculations showed that the current DIGE experiment requires a minimum of 10 samples from each group to be confident of 1.5 fold difference at the p<0.05 significance level. In a complementary targeted approach, Cytometric Beadarray (CBA) analysis showed no significant difference in the serum concentration of IL-6, IL-8, MIG, Eotaxin, IP-10, MCP-1, and MIP-1α, between clinical responders and non-responders, despite the association of these proteins with IFNβ treatment in MS.

## Introduction

Multiple sclerosis (MS) is a chronic, inflammatory, and demyelinating autoimmune disorder of the central nervous system (CNS). The disease course of relapsing remitting (RRMS) involves periods of clinical remission interspersed by exacerbations or relapses, which vary in their severity and duration. After many years of RRMS, the patient may enter a secondary progressive phase (SPMS) where the symptoms and nerve function gradually worsen with or without relapses. IFNβ treatment significantly reduces the frequency of relapses, lesion load, and disability in RRMS and SPMS patients [1], [2]. However, up to a third of patients do not respond to this therapy [3], [4]. Amongst them, a number of patients develop antibodies to IFNβ that prevent binding of the protein to its receptor (neutralizing antibodies: NABs), reducing or abrogating the therapeutic effect of IFNβ [5], [6]. It is important to identify people who do not respond clinically to IFNβ promptly, so they can be treated with less immunogenic IFNβ or alternate therapies at an early stage in the disease course [7], [8]. It has been shown that RRMS patients who are clinical responders to IFNβ treatment show a more inflammatory and less neurodegenerative disease at the commencement of the treatment compared to those who do not [9], however no specific biomarkers were found to differentiate these groups.

The aim of this study was to identify clinical response markers to IFNβ treatment in MS using proteomics. Discovery-driven and targeted protein approaches were used. Discovery driven proteomic approaches were first employed to generate the MS specific protein profile from cerebrospinal fluid CSF [10]–[12]. A 2D-DIGE approach was recently used to identify CSF markers in MS after comparison with other neurological disorders [13], [14]. 2D-DIGE was also used in a study to differentiate the CSF proteome of clinically isolated syndrome (CIS) patients that develop MS from the ones that remained CIS [15]. 2D-gel electrophoresis (2DGE) has been previously used to characterize the effect of serum from MS patients with and without IFNβ treatment on human cerebral endothelial cells [16]. However, to our knowledge, none of these techniques have previously been used in examining clinical response to IFNβ treatment in MS. We hypothesised that proteins which differ in abundance between the plasma proteome of clinical responders and non-responders to IFNβ could serve as clinical response markers for treatment with IFNβ. Furthermore, these proteins would be able to be identified using difference in-gel electrophoresis (DIGE) and mass spectrometry.

In any complex proteomic experiment, it is critical to determine the sample size required to provide sufficient statistical power to detect biologically significant changes [17]–[19]. Hunt *et al.* [18] established a protocol for optimizing experimental design. In this paper, we adopt this approach, using a relatively small sample size to enable accurate determination of statistical power while simultaneously identifying an initial set of putative biomarkers.

Another important aspect of a DIGE study is optimizing sample preparation to simplify the protocol and obtain a high resolution gel. To obtain that, we have compared different depletion and desalting techniques to remove the most abundant proteins and achieve better protein separation respectively.

The targeted approach was employed to identify lower abundant clinical response markers which may be undetectable by discovery driven approaches such as 2D-DIGE, either because more highly abundant proteins obscure their presence or because the removal of highly abundant proteins also depletes lower abundant proteins, like cytokines and chemokines, bound to these proteins [20]. In the targeted approach, we hypothesised that protein markers previously associated with IFNβ treatment, such as chemokines and cytokines, would also be markers of clinical response. In MS, chemokines act as chemoattractants: recruiting immune cells and the proinflammatory cytokines they secrete, such as IL-2, IFNγ, and TNFα, which promote processes leading to demyelination [21]. In addition they upregulate adhesion molecule expression which will assist in transendothelial migration of autoreactive immune cells through the blood brain barrier (BBB) [22]. IL-8 [23], [24], IP-10 [25], MCP-1 [26], [27], MIP-1α [28], and MIG [29] levels were previously found to be differentially expressed in sera from patients with IFNβ treated MS as compared to untreated MS patients. Although changes in serum Eotaxin are not known to be directly associated with IFNβ treatment, it is regulated by Th2 cell mediated cytokines [30], which play a significant role in the IFNβ treatment mechanism [31] is also found to be reduced in CSF from people with MS as compared to HC [32]. Th2 induced IL-6 was the only anti-inflammatory cytokine chosen for the current study, as its levels are increased in the serum of MS patients following IFNβ administration [33]. Despite their previous associations with IFNβ treatment, the status of these chemokines and IL-6 as clinical response markers to IFNβ is unknown and will be explored in this study.

## Materials and Methods

### Ethics approval

This study was approved by the Sydney West Area Health Service Human Research Ethics Committee (HREC2006/2/4.33(2310)).

### Sample collection

#### Discovery driven approach

Fresh blood was collected from 3 individuals who showed a clinical response to treatment (clinical responders - CRs) and 3 individuals who did not show a clinical response (clinical non-responders - CNRs) in P100 tubes (BD bioscience, NJ, US) 24 hours after IFNβ injection. P100 tubes were used to enable greater recovery and preservation of plasma proteins. Each group had two RRMS and one secondary progressive MS patient (SPMS); the response status was defined in different ways for each group. All six patients were under the care of a neurologist with special interest in MS and had definite MS by the McDonald criteria [34]. The RRMS CNRs (n = 2) had a history of at least one relapse in the last 2 years as compared to none for CRs (n = 2) since the commencement of their treatment (Table 1). The SPMS patient CNR (n = 1) showed an annual increase in the disability index (Expanded Disability Status Scale (EDSS) score) during the last year of IFNβ treatment while the SPMS CR (n = 1) had no change in EDSS (Table 1). In addition, MRI scans of the SPMS CNR revealed greater degree in progression of cerebral atrophy as compared to the SPMS CR during the last year of treatment. The tubes were centrifuged at 2500 *g* for 10 minutes at 20°C, within 2 hours of blood collection. The separated plasma was immediately aliquoted and stored at −80°C to prevent protein degradation. Individual aliquots were prepared to prevent freeze-thaw cycles.

**Table 1:** Demographics and clinical status of patients used in discovery-driven approach.

No.	Status	Gender	Age	Relapses before*	Relapses after*	EDSS before*	EDSS after	Type of MS	Type of treatment	Cy Dye
1	CR	F	46	0.4	0	-	-	RRMS	Avonex	Cy3
2	CR	F	28	1	0	-	-	RRMS	Betaferon	Cy5
3	CR	F	46	-	-	6	6	SPMS	Rebiff	Cy3
4	CNR	M	30	0.5	>1	-	-	RRMS	Avonex	Cy5
5	CNR	F	42	-	-	6	7	SPMS	Betaferon	Cy3
6	CNR	F	32	1	1	-	-	RRMS	Avonex	Cy5

Dyes were swapped for one CR and one CNR to remove dye bias.

*Relapses before and after are the annualised relapse rates before and after commencement of IFNβ treatment respectively for RRMS patients. EDSS before and after gives annual EDSS score before and after the last year of IFNβ therapy respectively for SPMS patients.

#### Targeted approach

Serum samples were obtained from 18 clinical responders (CR) and 19 clinical non-responders (CNR) to IFNβ treatment from diagnostic laboratories across Australia, as a part of a Neutralizing Antibody (NAB) testing service. 27 of those patients were negative for NAB (NAB–ve) while 10 were NAB-positive (NAB+ve). Patients gave informed consent for their neurologists to provide relapse histories for this study. The patients were classified as CR if they had no relapses during the previous year of treatment [3]. Otherwise, they were classified as CNR. Patient demographics are shown in Table 2. IFNβ treated patients with serum titres of 20 neutralizing units/ml or greater for two consecutive measurements at a 3 month interval using the cytopathic effect (CPE) bioassay (measures the serum concentration required to neutralize the antiviral activity of the IFNβ) were considered NAB+ve [35]. Otherwise they were considered NAB-ve. The samples were stored at −20°C until used. Serum was diluted 1∶4 for the chemokine analysis for optimal results.

**Table 2:** Demographics of patients providing samples for the targeted approach.

	CNR	CR
**Average age (years)**	48±11	44±13
**Gender ratio (F∶M)**	15∶4	13∶5
**Disease subtype (RRMS∶SPMS)**	11∶8	16∶2
**Average duration on treatment (years)**	4.5±2.2	3.5±2.2

### 2D- gel electrophoresis protocol optimization

Prior to 2D-DIGE, the process for 2D-gel electrophoresis was optimized. Several commonly used proteomic techniques were trialled in various combinations to identify the optimal technique for use in this study. These methods are presented in detail in A-F below. All the optimization steps were carried out using 11 cm IPG strips and 4–15% pre-cast SDS-PAGE gels (Bio-Rad Laboratories, CA, USA) except for method F. Protein concentration was estimated using the 2D-Quant kit following the manufacturer's instructions (GE Healthcare, Hercules, UK) before adding the reducing agent and IPG buffer 1% (v/v). IPG buffer (of the same pH as the strip) was added just before the rehydration step. 200 µg of sample was loaded onto each 11 cm gel (Method A–E) and 300 µg was loaded onto the 17 cm gel (Method F). Spot count was performed for each of the methods using the PDQuest v7.3.1 (Bio-Rad, CA, USA) spot detection module.

Method A) The sample was mixed with rehydration buffer (RB) (7 M Urea, 2 M thiourea, 4% CHAPS, 40 mM Tris) and 5 mM TBP, without a depletion and desalting step, followed by rehydration on pH 3–10 non-linear (NL) IPG strips (Bio-Rad Laboratories CA, US).

Method B) Albumin and IgG were depleted from the plasma using the Aurum serum protein mini kit according to manufacturer's instruction (Bio-Rad Laboratories, CA, US) followed by blending with RB +5 mM TBP. No desalting was undertaken. The sample was then rehydrated on a pH 3–10 NL IPG strip.

Method C) Aurum serum depletion was followed by a desalting process using the Khan *et al.* protocol [36]. In brief, the Aurum serum depleted sample was precipitated with ice-cold acetone at −20°C overnight and pelleted at 4000 *g* for 60 minutes at 10°C. The pellet was resuspended in RB with 5 mM TBP and 10 mM acrylamide for 60 minutes. The sample was spun at 21000 *g* to remove any insoluble impurities. The sample was then diluted with 13 ml RB without Tris, and added to 5 kDa 15 ml Amicon Ultra-15 centrifugal filters (Millipore MA, US). The protein solution was desalted and concentrated at 2000×*g* until the volume reached 350 µl. The desalted sample was then rehydrated on the pH 4–7 IPG strip.

Method D) Aurum serum sample depletion was followed by sample clean up using the 2D-clean up kit (GE Healthcare, Hercules, UK) following the manufacturer's instruction. This kit removes impurities such as nucleic acids, lipids, and salts. The clean pellet was resuspended in RB with 65 mM DTT for reduction for 30 minutes and 130 mM iodoacetoamide for alkylation for 30 minutes followed by rehydration on pH 4–7 IPG strip.

Method E) Aurum serum depletion was replaced by Multiple Affinity Removal System (MARS) (Agilent technologies CA, US) column depletion, undertaken at the Australian Proteome Analysis Facility (Sydney, Australia). This process removes the top six most abundant proteins (85%) in plasma, namely albumin, IgG, IgA, alpha-1 antitrypsin, transferrin, and haptoglobin. The dilute samples were applied to 5 kDa Amicon Ultra-15 centrifugal filters (Millipore MA) and spun at 2000×*g* to concentrate the solution to 400 µL. The sample was cleaned using 2D-clean up kit. The purified protein pellet was suspended in RB followed by a 30 minute reduction step with 65 mM DTT and 30 minute alkylation with 130 mM acrylamide. The sample was rehydrated on a pH 4–7 IPG strip.

Method F) The same process was used as in Method E except a 17 cm IPG strip was used instead of an 11 cm strip. The first and second dimension steps were generally consistent between the protocols (A–F).

### DIGE sample preparation and labelling

The samples, prepared as described in method F were labelled using fluorescent Cy Dyes as per manufacturer's Instructions (GE Healthcare, Hercules, UK). The sample labelling is shown in Table 1.

### 2D-Gel electrophoresis

IPG strips (pH 4–7, 11–17 cm, Bio-Rad Laboratories CA, USA) were rehydrated with the Cy Dye-labelled samples for 18 hours at room temperature. Iso-electric focussing (IEF) was performed for a total of 95,000 Vh for 17 cm strips and 70,000 Vh for 11 cm strips at 20°C using the IPGphor-II apparatus (GE Healthcare, Hercules, UK). Strips were equilibrated prior to SDS-PAGE for 15 minutes in equilibration buffer (6 M urea, 50 mM Tris pH 8.8, 20% (v/v) glycerol, 2% (w/v) SDS) with 5 mM TBP and then for 15 minutes in same equilibration buffer with 2.5% (v/v) acrylamide. The SDS PAGE was run by laying the strips on 8–18% large size (20 cm×20 cm) precast gels (Jule Inc., CT, US). The strips were overlaid with 1% agarose in SDS running buffer containing bromophenol blue. Tris-glycine (24.8 mM Tris, 192 mM glycine, 0.1% (w/v) SDS) running buffer was used. The 11 cm gels were run on a Criterion gel system (Bio-Rad laboratories CA, USA) for the 2^nd^ dimension at 200 V for 40 minutes or until the dye front ran off the bottom of the gel. The 17 cm gels were run at 16 mA/gel for one hour, 20 mA/gel for the next 3 hours, and 24 mA/gel thereafter until the bromophenol blue dye front had run off the bottom of the gels. Gels were scanned immediately. A non-DIGE preparative gel was run with 600 µg protein under the same conditions from a pool of all samples. This gel was stained with Coomassie blue and was used to excise spots for protein identification.

### Gel imaging

The protocol optimization gels were initially fixed in Fix/Wash Buffer (10% (v/v) methanol, 7% (v/v) acetic acid) for 1 hour followed by Sypro Ruby staining overnight. The gels were then washed with Fix/Wash buffer for 1 hour before scanning with Typhoon9410 scanner (GE Healthcare, Hercules, UK) following manufacturer's instructions. The DIGE images were also scanned using Typhoon scanner following manufacturer's protocol for DIGE images. Image analysis was performed using the DeCyder software package v5 (GE Healthcare, Hercules, UK), a 2-DE analysis software specifically designed for DIGE experiments. 2500 spots were identified and quantified using the DeCyder difference in-gel analysis (DIA) module. Proteins were deemed to be differentially expressed if they showed greater than 1.5 fold change in abundance between CR and CNR and p<0.05 using a 1-way ANOVA.

### Protein identification

Proteins of interest were excised from the preparative gel for protein identification. Preparation for protein identification was undertaken using the protocol described in *Gez S. et al.* [37] Peptide mass fingerprints of tryptic peptides were generated by MALDI-time of flight (MALDI-TOF) mass spectrometry using a Voyager DE-STR (Applied Biosystems, CA, US). The resulting data peaks were calibrated with trypsin autolysis peaks (842.51, 1054.56, and 2211.10 Da). The data was visualized using Data Explorer v4.5 (Applied Biosystems, CA, US) and analysed with Mascot (Matrix Science, London, UK) for protein identification comparing it with the MSDB and SWISS-PROT databases. Protein identifications were assigned to potential matches with an expectation value less than 0.01 and a score greater than 80. For higher molecular weight proteins, a more stringent expectation value (less than 0.001) was used. The spots identified by MALDI-TOF were confirmed using tandem mass spectrometry on a QSTAR (Applied Biosystems, CA, US) at the Sydney University Proteome Research Unit (SUPRU, University of Sydney, Sydney, Australia).

### Power calculations

An ongoing issue with complex proteomic experiments is the low statistical power of an experiment resulting in an inability to detect biologically significant changes. In the current study we used a process of experimental design to estimate the optimal sample size required to provide adequate statistical power at our chosen level of significance. Power calculations were conducted using tools provided by Emphron Informatics (www.emphron.com) according to the protocol defined by Hunt *et al* [18]. Breifly, volumes of 100 spots matched across all the six gels were selected for the analysis. These spots were randomly selected across the entire gel to remove any bias towards a particular region of the gel. The protocol estimates biological and technical variance between the groups using a mixed effects linear model involving a fixed term for the difference between groups and random deviations representing differences between samples and gels. Due to the use of the 2D-DIGE internal standard and the associated normalization between gels, the gel-gel difference was set to null. The software uses the estimated biological (sample) variation to determine the standard error of differences between CR and CNR before calculating the number of samples required to give 80% power (probability of not detecting a real change in protein expression) when attempting to find changes in expression of a given size at a given level of statistical significance (probability of falsely identifying a change in protein expression).

### Cytometric beadarray

Levels of IL-6, IL-8 (CXCL8), IP-10 (CXCL10), MIP-1α (CCL3), MCP-1 (CCL2), MIG (CXCL9), and Eotaxin (CCL11) in serum were determined in the chemokine analysis samples by utilizing a cytometric bead array (CBA) (BD Bioscience, NJ, US) kit and fluorescence detection by an LSR II flow cytometer (BD Bioscience, NJ, US). Briefly, 50 µl of beads with different fluorescence intensities and coated with cytokine or chemokine specific antibodies were added to 50 µl of diluted patient sera and incubated for an hour at room temperature in the dark. 50 µl of phycoerythrin-conjugated secondary antibody specific to cytokine or chemokine was added to the mixture and incubated for 2 hours at room temperature away from light. Simultaneously, standards for each cytokine or chemokine were treated in the same manner. Beads were washed to remove unbound detection antibodies and were analysed using a flow cytometer. Cytokine and chemokine concentrations were estimated using the CBA software. The statistical significance of the study was calculated using Wilcoxon's test with p-values of <0.05 considered statistically significant.

## Results and Discussion

### 2D-gel electrophoresis protocol optimization

In optimizing sample preparation protocols for gel electrophoresis, we seek to resolve the maximum number of spots and to obtain clear separation between these spots. This improves the accuracy of subsequent image and mass spectrometric analyses.

**Figure 1:**
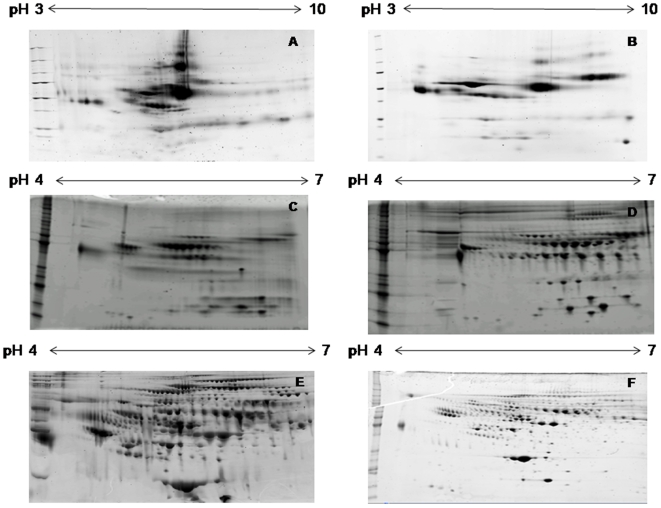
2DE gel images of human plasma samples generated with the different sample preparation methods used during optimization. Method A) Crude plasma - 224 spots, Method B) Aurum serum depleted plasma without desalting– 86 spots, Method C) Aurum serum depleted plasma desalted according to *Khan et al.* protocol– 78 spots, Method D) Aurum serum depleted plasma cleaned up using 2D-clean up kit and alkylated using iodoacetamide - 212 spots Method E) MARS depleted plasma followed by 2D-clean up and alkylated using acrylamide– 774 spots, Method F) 17 cm gel with same protocol as E– 1041 spots. Each ladder had molecular weight ranging from 10 to 220 KDa.

In method A (Figure 1A), only the most abundant proteins such as albumin, haptoglobin, fibrinogen, IgA, and IgG are clearly visible. To maximize the number of spots seen on the gel we depleted these highly abundant proteins, as they masked the presence of less abundant proteins and interfered with their resolution on the gel. We compared two depletion techniques. After Aurum serum depletion of albumin and IgG which constitute 70–80% of the total proteins in plasma (method B–D), we found the spot resolution was better and display of less abundant proteins increased as compared to method A. However, the number of spots remained the same or decreased due to protein loss (Figure 1A–D). Also, Aurum serum depletion uses resin blend columns which do not induce absolute removal of the two proteins; and over 10% of these proteins remain in the sample. (Figure 1B–D). In Method E and F, the MARS column depletion showed a further 3–4 fold increase in the number of spots as compared to Aurum serum depletion (methods B–D, Figure 1E–F). Depletion with MARS column resulted in removal of approximately 99% of the top six most abundant proteins which constitute 85–90% of total proteins in plasma.

In method A and B (Figure 1A and B), horizontal streaking between the spots is visible, indicating lack of protein separation. Clear separation between protein spots can be achieved by removing salts and charged contaminants as these affect iso-electric focussing. In the current experiment we compared two different desalting techniques. The first procedure incorporated desalting via acetone precipitation followed by diafiltration or buffer exchange [36] (method C). The separation of spots was better than method A and B but was not uniform across the gel despite the desalting step. This may be attributed to higher conductivity and low yield of protein prevented further rounds of buffer exchange as it would decrease the conductivity at the cost of higher protein loss. The second desalting procedure uses a 2D-clean up kit (Figure 1D–F). Despite using similar concentration to begin with, the protein concentration after 2D-clean up (1.2 mg/ml) was high compared to the buffer exchange method (0.73 mg/ml) indicating the former results in lesser protein loss. The protein separation was enhanced, protein loss was minimized, and desalting efficiency was improved with the 2D-clean up kit as compared to the single buffer exchange method.

In Method D (Figure 1D), horizontal streaking was completely removed, except in some parts of the gel at the acidic end which showed aggregate formation during the iso-electric focussing step. This issue was eliminated by the use of acrylamide as an alkylating agent instead of iodoacetamide before iso-electric focussing as shown in (Figure 1E). Iodoacetamide may induce cross reactivity with thiourea which could have caused improper alkylation, leading to reformation of disulfide bridges and hence the streaking at positive end [38]. Thus, we found acrylamide to be a better alkylating agent than iodoacetamide.

Finally, in method F, the 17 cm gel showed a better separation of protein spots due to larger gel size (Figure 1F). Thus, it was determined to be the most appropriate technique for of depleting abundant proteins, desalting, and alkylation of the sample before isoelectric focussing ensured a lower protein loss resulting in a greatly increased number of spots displayed on a 2D-gel image with proper separation to help with further analysis and spot selection. It was this method which was used in the preparation and running of the DIGE gels.

### Power analysis

**Figure 2:**
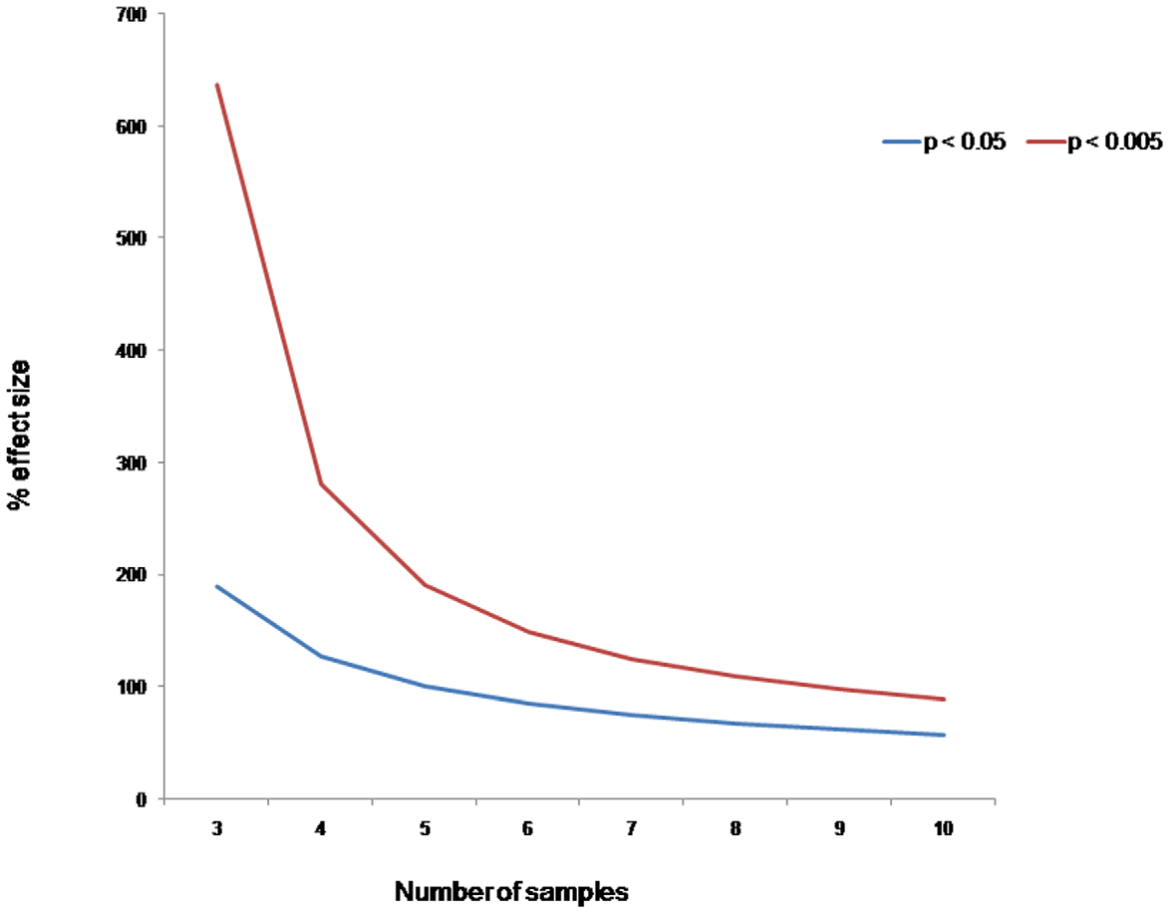
Power curve showing the minimum % effect size (fold change) detectable as a function of sample size with 80% power at two different significance levels.

The primary aim of the discovery driven approach was to produce sufficient 2D-DIGE data to enable statistical power calculations to determine optimal sample size. DIGE studies use an internal standard to help eliminate gel-to-gel variation [39]. However, normal biological variation between the samples remains and this can lead to false conclusions as this variation interferes with the ability to detect variation between the groups of interest. Proteomic studies require sufficient statistical power to overcome these sources of variation. To address this issue, we conducted power calculations based on the modified protocol of Hunt *et al* [18]. To our knowledge this is first DIGE study which incorporates power calculations to determine optimal sample size. The power analysis tool was used to calculate the minimal detectable difference defined as the size of effect required to give a chosen statistical power at a specific significance level. The results are shown in Figure 2. The power calculation showed that a minimum of 10 biological variants from each group are required to be confident of a 1.5-fold (50% effect size) and 2 fold (100% effect size) change in abundance between CR and CNR at the p<0.05 and p<0.005 level of statistical significance respectively.

### Identification of differentially abundant proteins

The secondary aim of the discovery driven approach was to identify differentially abundant proteins detectable with the existing sample size. The power analysis indicated an optimal sample size of 10. This means ten samples per groups are required in order to be reasonably certain of detecting statistically significant (p<0.05) changes in protein expression at least 1.5 fold in size. However, it still remains possible, albeit less likely, to find statistically significant changes using smaller sample sizes, particularly if the fold-change is greater than 1.5. In statistical terms, the use of a smaller sample size does not affect Type I error (falsely deciding a protein is differentially expressed) but will increase Type II error (not detecting proteins that are actually differentially expressed). As the three-sample-per-group analysis was required in order to conduct the power calculations, we chose to explore this data for the presence of an initial set of potential biomarkers.

**Figure 3:**
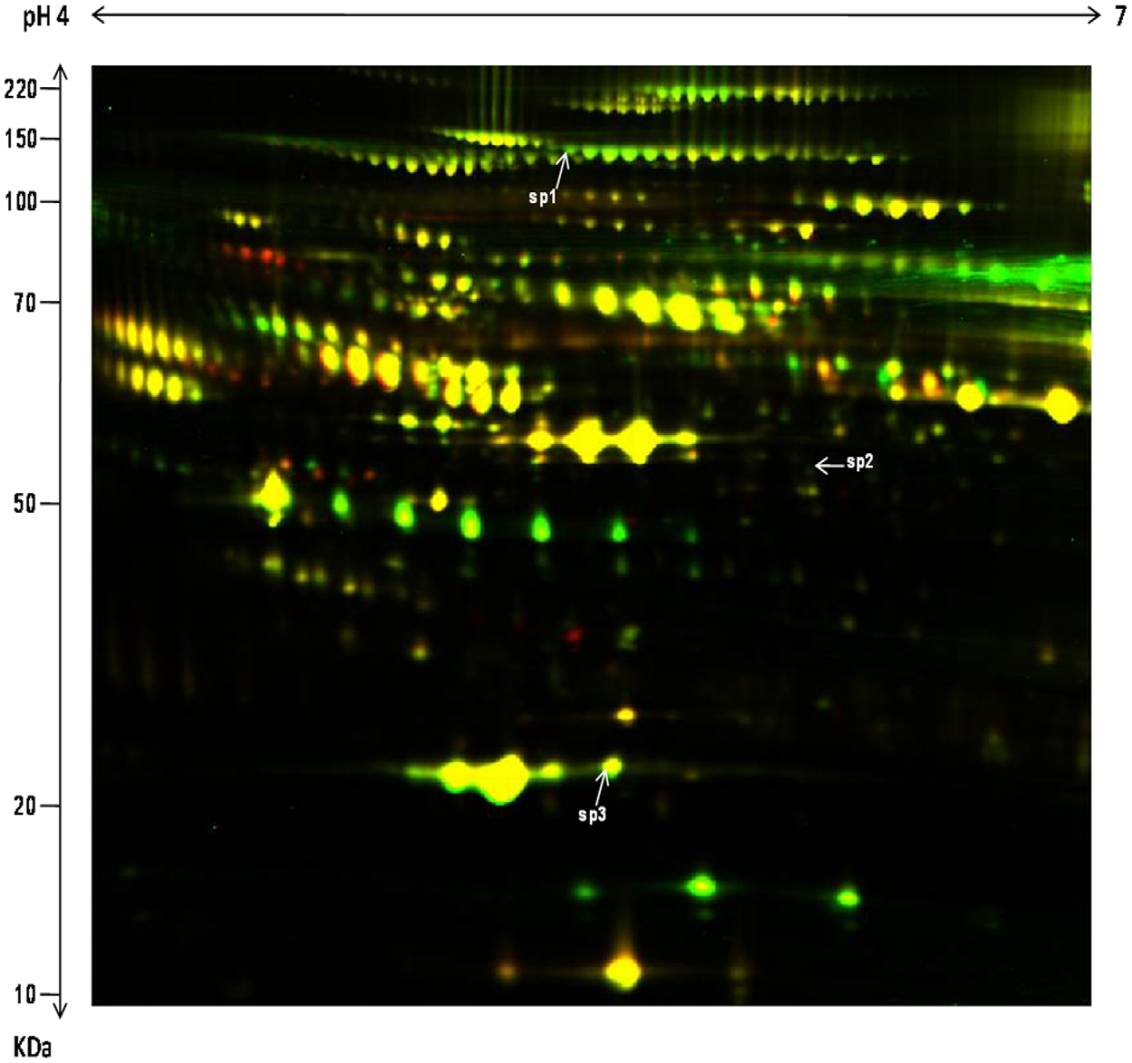
A 2D-DIGE image comparing the plasma proteome of CR to IFNβ treatment with CNR to IFNβ treatment with Cy3 and Cy5 channel overlap. Samples were run on 17 cm gels after clean up and depletion. Spots Sp1, Sp2, and Sp3 were found to be increased in CR as compared to CNR based on a 1-way ANOVA with a p-value of less than 0.05 and fold change greater than 1.5.

The DeCyder biological variation analysis (BVA) module was used to match spot maps and conduct statistical analysis to identify differentially expressed spots based on the standard abundance value for each spot. Three spots were found to be more abundant in clinical responders to IFNβ as compared to non-responders with a fold difference of at least 1.5 at the p<0.05 level of significance (Figure 3) (Table 3). These spots were identified as Alpha 2 macroglobulin (A2M), Apolipoprotein A1 (APOA1), and Fibrinogen B (FIBB) using MALDI-TOF mass spectrometry (Figure 3) (Table 3). The protein identification was further confirmed using tandem mass spectrometry where three peptides were matched for each protein.

**Table 3:** Differentially expressed spots between CR and CNR with p-value <0.05 and fold change >1.5.

No	Fold	Protein	p	Score	E-value	Match	mW/pI
sp1	2.12	Alpha-2 macroglobulin	0.034	88	3.1×10^−5^	12	164951/6.00
sp2	2.1	Fibrinogen beta chain	0.025	86	4.1×10^−5^	12	56745/8.54
sp3	1.72	Apolipoprotein A1	0.049	124	7.2×10^−9^	11	30759/5.56

No is the spot number; Fold is the fold change in expression; p is the p-value associated with the fold change in expression; Score and expectation value (E-value) indicate the confidence of protein identification; Match is the number of matching peptides; mW/pI is the theoretical molecular weight and isoelectric point as calculated from Mascot database; all spots were confirmed with tandem mass spectrometry.

A2M is a known inhibitor of matrix metalloproteinase activity (MMP2 and MMP9) along with their other tissue inhibitors (TIMP1 and TIMP2) [40]. Previous studies have shown no difference in the plasma level of total A2M between MS and HC [41], [42] however both MMP9 and the MMP9:TIMP1 ratio in the serum of MS patients are high compared to HC [43] while these levels are reduced in patients treated with IFNβ [44]. A recent study showed that TIMP-1 levels increased longitudinally in clinical responders to IFNβ treatment as compared to clinical non-responders [45]. Together with our finding that MMP inhibitor A2M levels are increased in the plasma of CRs compared to CNRs, the data suggest that matrix metalloproteinase inhibitors may prove useful as clinical response markers to IFNβ treatment in MS. A2M has previously been found to be downregulated in CSF in a study comparing RRMS with other neurological diseases and a separate study comparing HIV-1 with and without dementia [46]. This suggests any pathogenic link with MS may not be specific. Functional studies, such as phenotype characterisation studies, would help address this question.

APOA1 levels were significantly lower in plasma at the end of the first year of treatment in patients treated with IFNβ, who suffered relapses after therapy or progressed in their EDSS score, as compared to their basal values [47]. While there was no significant difference between the CRs and CNRs, there was a trend towards down regulation in non-responders, which concurs with our analysis. APOA1 acts as an inhibitory factor for proinflammatory cytokines IL-1β and TNFα that have been suggested to have a role in MS pathogenesis [48]. The same research group also showed IFNβ inhibits the ability of T-cells to induce IL-1β and TNFα [49]. Thus IFNβ may induce APOA1 which inhibits the activity of proinflammatory cytokines in CRs. APOA1 is also responsible for the inhibition of adhesion molecules ICAM-1 and VCAM-1 [50], that assist in auto-reactive T-cell extravasation through the blood brain barrier (BBB) in MS pathogenesis [51]. Thus, APOA1 is a putative clinical response marker to IFNβ treatment that should be further validated.

To our knowledge, there is no known association between FIBB and IFNβ treatment or FIBB and MS.

Timms and Cramer recently reviewed the utility of DIGE as a quantitative technique [52]. They found DIGE has a number of strengths as a quantitative technique but also certain limitations. Furthermore, this balance of strength and limitation was held in common with other quantitative approaches, suggesting the various techniques are complementary. Thus, a follow-on study, using the optimal sample size determined in this study, and incorporating validation with a second quantitative method, is desirable to confirm the status of the differentially expressed proteins identified in this study as putative clinical response markers.

### Cytometric Bead Array analysis

**Figure 4:**
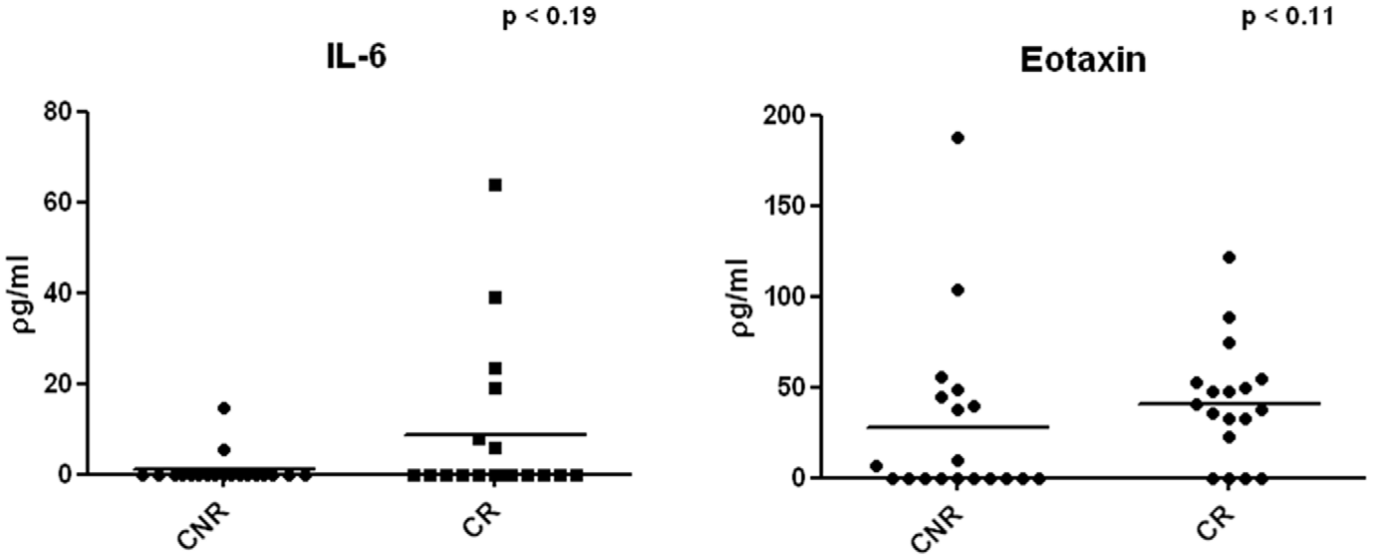
IL-6 and eotaxin concentration comparison between CR and CNR in serum.

IL-6 (p = 0.2), IL-8 (p = 0.53), MIG (p = 0.3), MCP-1 (p = 0.61), eotaxin (p = 0.11), IP-10 (p = 0.81), and MIP-1α (p = 0.61) serum concentrations were not significantly different between CR and CNR to IFNβ. However, both eotaxin and IL-6 levels were high in CR as compared to CNR, but not significantly different, partly because some of the samples were below the level of detection (Figure 4). Eotaxin is produced by Th2 cells [53] and has previously been found to be reduced in serum of MS patients as compared to HC [32]. Hence a trend towards elevation of serum eotaxin in CRs could be an effect of Th2 bias generated by IFNβ treatment. CR also showed a trend towards a higher percentage of samples with detectable serum IL-6, another Th2 cytokine. Interestingly, increased IL-6 in IFNβ treated patients has been found to be associated with lower relapse rates and disability [54].

There was also no significant difference in the serum concentrations between NAB+ve and NAB-ve patients of any of the analytes: IL-6 (p = 0.99), IL-8 (p = 0.43), MIG (p = 0.92), MCP-1 (p = 0.77), Eotaxin (p = 0.33), IP-10 (p = 0.23), and MIP-1α (p = 0.21).

### Concluding remarks

In summary, we identified an optimal depletion and desalting technique for gel-based plasma proteomics in clinical samples from MS patients. The pilot DIGE experiment identified A2M, APOA1, and FIBB as putative clinical response markers for IFNβ treatment. This study for the first time incorporates power calculations in a 2D-gel approach to determine the optimal sample size required to be confident to characterize clinical response markers. The targeted approach suggests that eotaxin and IL-6 require further investigation as possible IFNβ clinical response markers.

Both discovery-driven and targeted approaches demonstrated a requirement for a larger sample size to fully characterize clinical response markers using a proteomic approach. Furthermore, heterogeneity in multiple sclerosis itself, as well as the approaches to treatment with IFNβ, also suggests the need for larger sample sizes to help eliminate others sources of biological variability not directly related to the effect of IFNβ treatment. Thus, while we have identified an initial set of putative biomarkers, there are almost certainly more to discover through future studies involving larger sample sizes. This may increase the detectable differences in the protein profile between CR and CNRs, which may help identify more putative biomarkers, and improve our understanding of the mechanism of action of IFNβ in the treatment of multiple sclerosis.
